# Radiographic parameters and a novel fluoroscopic control view for posterior screw fixation of coracoid base fractures: A cadaveric study

**DOI:** 10.1051/sicotj/2020008

**Published:** 2020-05-05

**Authors:** Hatem Galal Said, Tarek Nabil Fetih, Hosam Elsayed Abd-Elzaher, Simon Martin Lambert

**Affiliations:** 1 Professor, Orthopaedics and Traumatology Department, Assiut University 71515 Assiut Egypt; 2 Assistant lecturer, Orthopaedics and Traumatology Department, Assiut University 71515 Assiut Egypt; 3 Assistant lecturer, Orthopaedics and Traumatology Department, Al-Azhar University 11651 Assiut Egypt; 4 Consultant, Department of Trauma and Orthopaedics, University College London Hospitals NHS Foundation Trust WC1E6BT London UK

**Keywords:** Coracoid base, scapular fractures, coracoid screw, coracoid view, shoulder trauma

## Abstract

*Introduction*: Coracoid fractures have the potential to lead to inadequate shoulder function. Most coracoid base fractures occur with scapular fractures and the posterior approaches would be utilized for surgical treatment. We investigated the possibility of fixing the coracoid through the same approach without an additional anterior approach. *Materials and methods*: Multi-slice CT scans of 30 shoulders were examined and the following measurements were performed by an independent specialized radiologist: posterior coracoid screw entry point measured form infraglenoid tubercle, screw trajectory in coronal plane in relation to scapular spine and lateral scapular border, screw trajectory in sagittal plane in relation to glenoid face bisector line and screw length. We used the results from the CT study to guide postero-anterior coracoid screw insertion under fluoroscopic guidance on two fresh frozen cadaveric specimens to assess the reproducibility of accurate screw placement based on these parameters. We also developed a novel fluoroscopic projection, the anteroposterior (AP) coracoid view, to guide screw placement in the para-coronal plane. *Results*: The mean distance between entry point and the infraglenoid tubercle was 10.8 mm (range: 9.2–13.9, *SD* 1.36). The mean screw length was 52 mm (range: 46.7–58.5, *SD* 3.3). The mean sagittal inclination angle between was 44.7 degrees (range: 25–59, *SD* 5.8). The mean angle between screw line and lateral scapular border was 47.9 degrees (range: 34–58, *SD* 4.3). The mean angle between screw line and scapular spine was 86.2 degrees (range: 75–95, *SD* 4.9). It was easy to reproduce the screw trajectory in the para-coronal plane; however, multiple attempts were needed to reach the correct angle in the parasagittal plane, requiring several C-arm corrections. *Conclusion*: This study facilitates posterior fixation of coracoid process fractures and will inform the “virtual visualization” of coracoid process orientation.

## Introduction

Fractures of the coracoid process are uncommon, comprising approximately 13% of all scapular fractures and 5% of all shoulder fractures [1, 2].

Coracoid fractures are usually seen with other fractures of the scapula or shoulder region; isolated coracoid fractures are rare [3].

Two classification systems exist for coracoid fractures. The Eyres classification comprises five groups on the basis of location: the type 3 basal fracture is the commonest [4].

The Ogawa classification has two groups based on relation to the coracoclavicular ligaments 2. Most coracoid fractures can be treated successfully non-operatively when none or minimally displaced [5, 6].

Unstable coracoid base fractures associated with other scapular fractures have the potential to lead to inadequate shoulder function [7–11].

When operative treatment is indicated simultaneous reduction and fixation of coracoid base fracture via an open or mini-open approach or percutaneously under fluoroscopic guidance is the surgery of choice with a lag screw application.

If an associated glenoid and/or scapula neck/body fracture is being treated through a posterior Judet approach, then indirect reduction of the superior glenoid with the attached coracoid can be accomplished. Speciﬁcally, in certain common fracture patterns such as the Eyres fracture types IV–VI, the superior glenoid fragment is attached to the coracoid, and when associated with a displaced scapula neck fracture, the preferred approach may be posterior depending on the size of the fragment and location of the fracture line. The modiﬁed Ideberg III glenoid fracture ﬁts this same description.

In such cases, ﬁxation rendered from the posterior approach indirectly ﬁxes the coracoid. Screws that can be most effective for this fracture variant begin on the acromial spine and are directed down into the scapular neck, ﬁxing the superior coronal fragment to its inferior host.

Otherwise, the coracoid can be addressed through a separate anterior glenohumeral approach, which is more invasive and technically difficult in the prone position. Even through an open approach, accurate screw placement into the coracoid base is difficult due to the complicated three-dimensional anatomy of the coracoid process [11, 12].

Since most coracoid base fractures occur with scapular fractures and the posterior Judet or Brodsky approaches would be utilized, we investigated the possibility of fixing the coracoid through the same approach, thus not requiring an additional anterior approach.

The primary purposes of this study were to describe an entry point, the trajectories (sagittal and coronal inclination angles), and an average length for a coracoid base-body lag screw applied from a posterior Judet approach. These values were then tested in a secondary pilot cadaveric study to evaluate proof of the principle. A novel imaging technique was developed to facilitate drill guidance and screw placement.

## Patients and methods

Multi-slice CT examinations of 30 shoulders of patients admitted to our hospital between April and December 2017 were examined. They underwent CT investigation for chest injuries that did not involve the shoulder girdle. The study involved 15 (50%) right and 15 (50%) left shoulders. There were 10 (33%) female and 20 (67%) male patients. Mean age was 45 years (20–70 years) in females and 42.5 years (20–65 years) in males.

Axial CT (Philips Brilliance 64) images were obtained using 1.25-mm thickness slices with bone window setting. Multiplanar reformats were created in Brilliance Workspace (Philips Medical Systems). Measurements were performed by an independent specialized radiologist.

**Figure 1:**
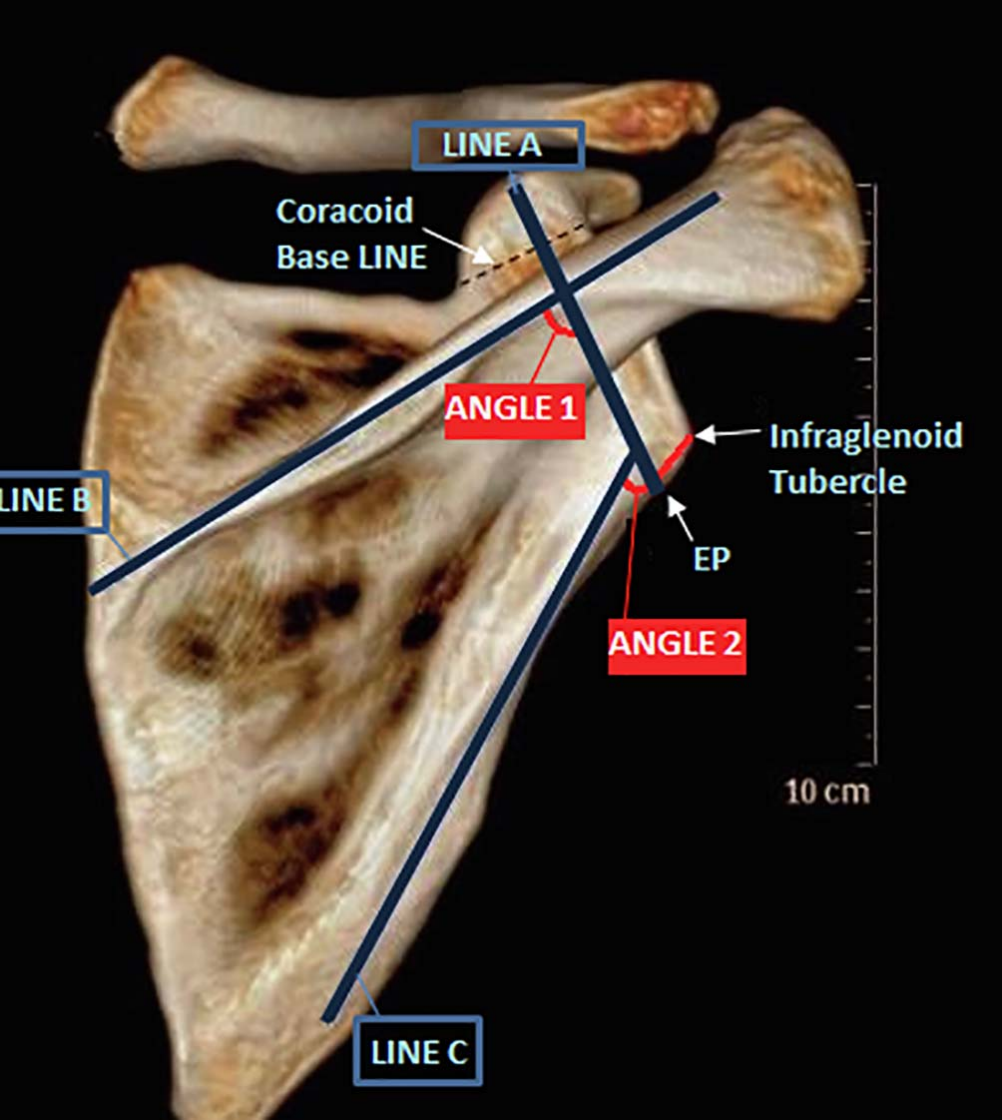
Coronal plane measurements. Entry Point (EP) is the point where line A which bisects the basal coracoid line and extends perpendicular to it intersects the inferior margin of the scapular neck. The distance from this point to the infraglenoid tubercle was measured in millimeters. The screw trajectory was determined by angle 1 between line A and line B (the long axis of the scapular spine) and angle 2 between line A and line C (the lateral scapular border).

The scapula-coracoid screw entry point (EP) was defined in the coronal plane as the point where a line (line A) which bisects the basal coracoid line and extends perpendicular to that line intersects the inferior margin of the scapular neck. The distance from this point to the infraglenoid tubercle was measured in millimeters (Figure 1).

The screw trajectory in the coronal plane (on an anteroposterior scapular projection, this approximates to a medial inclination) was determined as the angle between two lines of reference (Figure 1). The first angle was between line A and line B (the long axis of the scapular spine). This was considered to be a practical guide in an intra-operative situation. The second angle was between line A and line C (the lateral scapular border).

This was considered to be a practical guide in more extensile exposures of the scapula.

**Figure 2:**
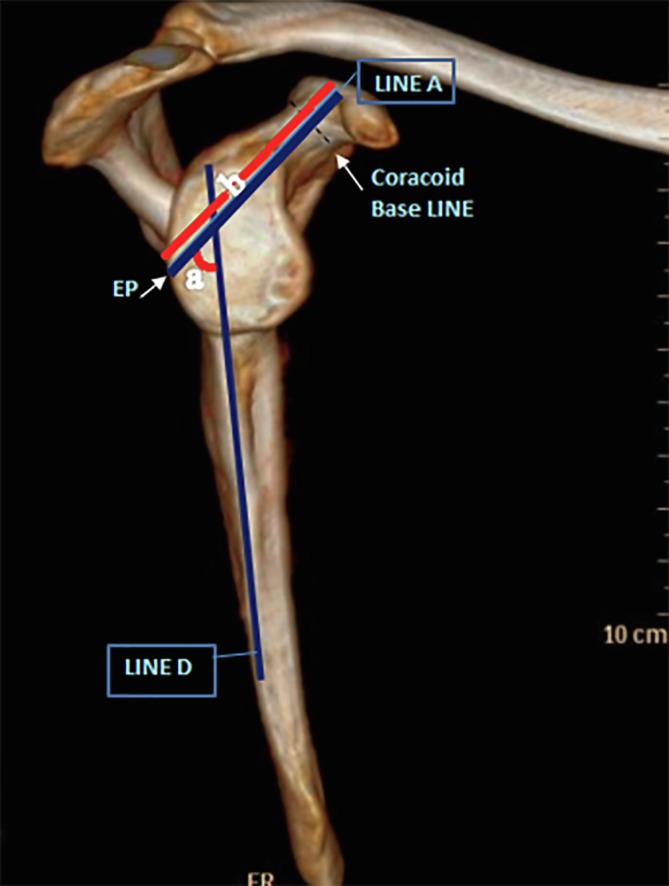
Sagittal plane measurements. (a) Screw angle between line A and line D (the glenoid face bisector line). (b) Screw length.

The screw trajectory in the sagittal plane (on a scapular-Y view, this approximates to an anterior inclination) was determined by the angle between line A and line D (the glenoid face bisector line), Figure 2.

The screw length was measured from the EP to the cortical exit point of a line which bisects the coracoid on the sagittal plane view (this approximates to line A, but is not the exact equivalent), Figure 2.

### Statistical methods

Mean and standard deviation the variables were calculated using SPSS software (version 18).

### Pilot cadaveric study

**Figure 3:**
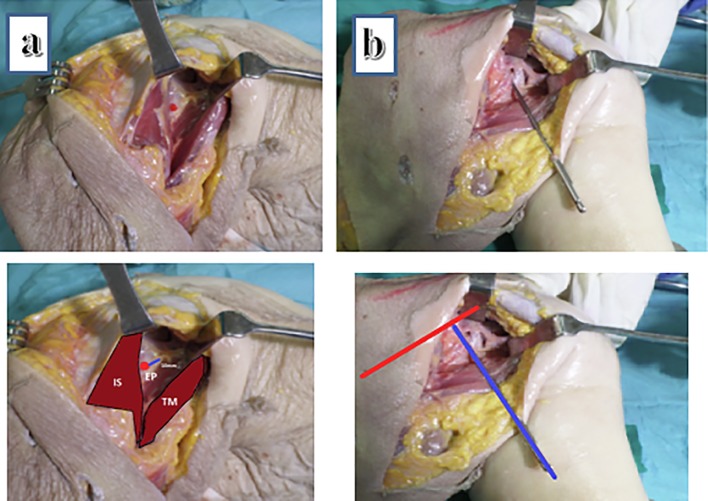
Cadaver specimen trial of screw application (Right shoulder specimen). (a) Entry point (EP), being the mean of all CT measurements, marked on the posterior scapular neck (red dot) 10 mm from glenoid rim (blue line) through a posterior surgical (Brodsky) approach between infraspinatous (IS) and teres minor (TM) muscles. (b) Ideal drill bit trajectory (blue line) in coronal plane referencing the para-coronal angle from scapular spine (red line) while the sagittal plane control is done under fluoroscopic guidance.

We used the results from the CT study to guide postero-anterior coracoid screw insertion under fluoroscopic guidance on two fresh frozen cadaveric specimens to assess the reproducibility of accurate screw placement based on these parameters (Figure 3).

We developed a novel fluoroscopic projection, the anteroposterior (AP) coracoid view, to guide screw placement in medial inclination (in the para-coronal plane).

**Figure 4:**
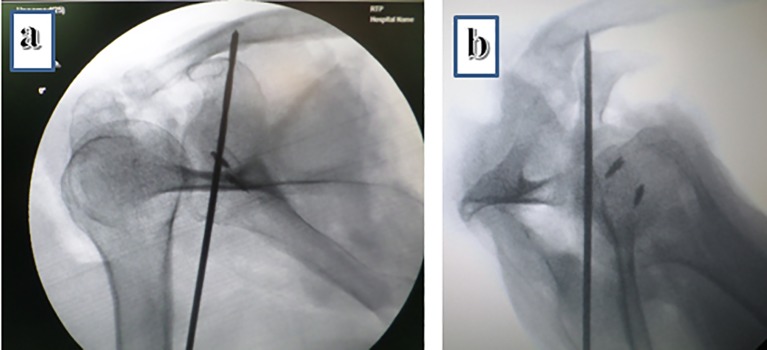
Fluoroscopic control of screw position. (a) Ideal screw position in coracoid base view. (b) Ideal screw position in scapular Y view (images showing anchors previously implanted in cadaver specimen)

With the shoulder girdle placed in the typical prone position, a standard C-arm was placed over the shoulder and inclined 45 degrees in a lateral to medial direction, and 25 degrees in a caudal to cranial direction until a clear AP view of the maximum width of the coracoid base was visualized. This view controlled screw placement in the coronal plane. We also speculated that reduction of a displaced coracoid base fracture by “joy-stick” manipulation would be facilitated by this view. For control of the screw position in the parasagittal plane, a classic scapular Y view could be used (Figure 4).

Ethical approval was obtained for this study from the Faculty of Medicine Ethical Committee of the University of Assiut. The patients were informed of the purpose of the study, consent was obtained for the use of patient data for the study, and all information was used in accordance with the requirements of confidentiality.

## Results

Thirty shoulders were evaluated for the CT study.

*Entry point*: The mean distance between the planned entry point and the infraglenoid tubercle was 10.8 mm (range: 9.2–13.9, *SD* 1.36).

*Screw length:* The mean screw length was 52 mm (range: 46.7–58.5, *SD* 3.3).

*Sagittal plane angle*: The mean sagittal inclination angle between screw line (line A) and glenoid face bisector line (line D) was 44.7 degrees (range: 25–59, *SD* 5.8).

*Coronal plane angle*: The mean angle between screw line (line A) and lateral scapular border (line C) was 47.9 degrees (range: 34–58, *SD* 4.3).

The mean angle between line A (screw line) and scapular spine (line B) was 86.2 degrees (range: 75–95, *SD* 4.9).

### Results of the pilot cadaveric trial

**Figure 5:**
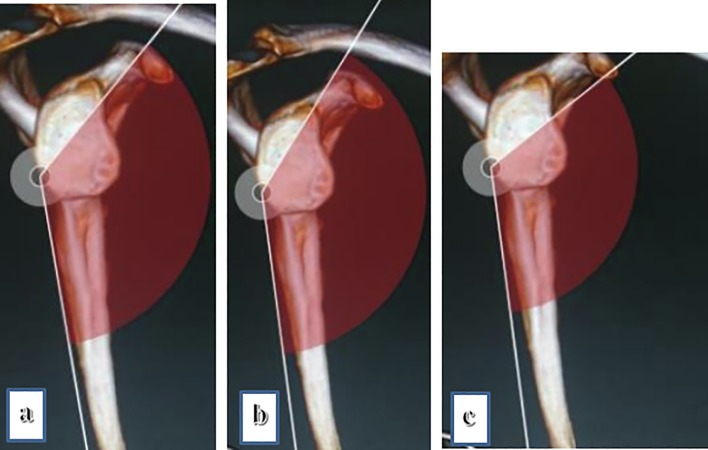
Effect of 10 degrees increase (b) and decrease (c) in sagittal inclination from the same entry point on final screw position relative to the proper mean inclination angle (a).

It was easy to reproduce the screw trajectory in the para-coronal plane, within 1–2 trials, especially utilizing the scapular spine as the reference line with an approximate average angle of inclination of 90 degree. However, multiple attempts were needed to reach the correct angle in the parasagittal plane, requiring several C-arm corrections. On measuring the variation, a change of only 10 degrees superior or inferior, resulted in a screw trajectory outside the coracoid (Figure 5).

## Discussion

The treatment of coracoid fractures is based on the site and degree of displacement of the fracture [2, 11–15]. Fractures of the coracoid base associated with displaced scapular neck and/or glenoid fracture are generally treated by open reduction and internal fixation of both fractures. Anterior open, mini-open, and fluoroscopically guided percutaneous techniques have been described for coracoid fixation [2, 14, 15]; however, screw application across the coracoid base is difficult due to the complex anatomy of the coracoid process [12, 13].

Ogawa et al. describe open reduction of the coracoid base fracture and internal fixation using a malleolar screw [2]. Duan et al. performed an open technique under fluoroscopic guidance which required an open anterior approach and placement of two guide wires and the position of those wires was confirmed by plain X-ray before placement of screws [14] Bhatia et al., described percutaneous coracoid fixation using two specific fluoroscopic guidance techniques to show the two coracoid pillars and the fluoroscopy beam was adjusted to obtain optimal visualization before any intervention [15]. These techniques require the addition of at least a limited anterior deltopectoral approach for inserting the coracoid screw when performing open reduction and internal fixation of associated scapular fractures via posterior surgical approaches: this can be difficult if the patient is positioned in prone. Our study aimed at establishing reference values for posterior coracoid screw application through the same posterior Judet or mini-open approach with regard to entry point and bi-planar insertion.

Although anatomic and radiographic measurements of the coracoid process have been reported previously [11, 12] there are no studies using CT axial measurement of the coracoid base. In order to insert a screw into the coracoid base accurately from posterior without complications (inadvertent brachial plexus or subclavian vessel injury), it is important to be able to “visualize” the cross-section and size of the coracoid base, and its spatial relationships when aiming from posteriorly.

The posterior-anterior coracoid screw insertion eliminates the need for adding an anterior approach to the already extensile posterior approach with the patient prone.

The CT study of the entry point and two plane trajectory angles for screw application in the cadaver trial part of the study, revealed that the entry point and paracoronal plane angle are reliable for accurate screw placement. The parasagittal plane reference angle appeared to be relatively poorly reproducible without fluoroscopic guidance. We think this is due to the entry point lying on the convex scapular surface: a small change of the position of the entry point on the curved surface may result in the coracoid base being missed even if the inclination angle was chosen correctly. Thus we recommend that the scapular Y view (under C-Arm guidance) should always be utilized to guide the screw accurately in the parasagittal plane. We have described a novel fluoroscopic view used in the pilot cadaver trial which can confirm coracoid base alignment and ensure screw placement through the coracoid base while eliminating the need for special surgical devices or techniques.

We chose the reference lines and points for assessment of inclination angles for practical intra-operative utility: it is helpful to define points of interest (e.g. the infraglenoid tubercle) within a reasonable surgical field-of-view, while improving accuracy of orientation by defining reference lines outside the field of dissection. This technique would lend itself to computer-navigation in future studies.

The intraoperative determination of orientation for drilling to distant, and hidden, targets is difficult. This study suggests that a “rule-of-thumb” reference for postero-anterior coracoid fixation, corrected by intraoperative imaging, might include a 45 degree medial inclination (in relation to the lateral scapular border) and a 45 degree sagittal inclination, noting greater variability in the latter, using a 50-mm screw from an entry point approximately 10 mm from the infraglenoid tubercle. The magnitude of these measurements, and their potential errors, are familiar to surgeons.

We believe that the entry point and paracoronal plane angle reference values can be used reliably and can be confirmed by the fluoroscopic view while the parasagittal plane angle is affected by the slightest change in entry point: it is less reliable and should be controlled by the fluoroscopic lateral Y view. This study demonstrates the anatomic and radiographic basics for the posterior coracoid screw fixation technique practice, further studies to accurately assess the reproducibility of an accurate screw placement during surgery are suggested.

## Conclusion

We believe that the data from this study will inform the “virtual visualization” of coracoid process orientation and coracoid basal fracture fixation using posterior surgical approaches for associated scapular fractures. Furthermore, this study facilitates fixation of coracoid process fractures while eliminating the need for an additional anterior surgical approach.

## Conflict of interest

The authors declare that they have no conflict of interest.
